# The Treatment of Tuberculous Peritonitis

**Published:** 1902-07-19

**Authors:** 


### THE TREATMENT OF TUBERCULOUS PERITONITIS.

In a lecture on Tuberculous Peritonitis delivered at Guy's Hospital, Dr. Hale White [1] recently gave some useful hints as to the choice of case for operation. In regard to the operation itself all authorities are agreed that laparotomy is much more reliable than puncture. Some surgeons, he says, wash out the abdomen with boiled water, others use weak antiseptics, others dust iodoform on some parts, others excise caseous nodules, and some take away the Fallopian tubes if the genital organs are tuberculous; "but as all surgeons appear to get equally good results, it seems clear that the chief point is merely opening the abdomen." As to whether to operate or not in any given case he points out that one should never operate if the peritonitis is merely a part of an acute general tuberculosis. Next, operation must not be done if there is already a fæcal fistula, for the presence of this condition shows that there must be numerous adhesions, and that one will in all probability not be able to get into the peritoneal cavity at all. It must be remembered that the best results are obtained in cases in which there is most fluid present. These are the very cases which do best whether operated on or not. Still, they are just the cases in which the risk of operation is exceedingly small, so that it is probably wise to open the abdomen and let the fluid out, especially if it be impossible to send the patient away into the fresh air. The best thing to do would probably be to open the abdomen, let out the fluid, and then send the patient away into the country. The worst results are obtained when there is little or no fluid in the abdomen; in these cases, therefore, the operation should not be done. Localised collections should be dealt with on the same principles. Localisation, however, points to the presence of adhesions, so one should be chary of touching them. The prominent umbilicus that is so often met with in these cases must never be incised. It often means that there is a tuberculous abscess under it which will sooner or later open into the bowel, and if it has been previously opened externally one will then have a fæcal fistula to deal with. Finally, if there should be a fæcal fistula one must not on any account try to deal with it by operation. The most disastrous results sometimes follow, and Dr. Hale White has never seen any good done by such attempts.

[1] Guy's Hospital Gazette, June 21.

